# The Use of 3D Printing Technology in Rehabilitation for Adults Living With Neurological Conditions: Scoping Review

**DOI:** 10.2196/81782

**Published:** 2026-05-06

**Authors:** Salena Aggerwal, Zahra Lalani, Jennifer O’Neil

**Affiliations:** 1 Faculty of Health Sciences, School of Rehabilitation Sciences, University of Ottawa, 200 Lees Avenue, Ottawa, ON, K1S 5S9, Canada, 1 6135625800; 2 Bruyère Health Research Institute, Ottawa, ON, Canada

**Keywords:** 3D printing technology, neurorehabilitation, rehabilitation technology, motor recovery, assistive devices

## Abstract

**Background:**

Neurorehabilitation plays a key role in improving motor recovery for people with neurological conditions. Although 3D printing has emerged as a promising rehabilitation tool, little is known on how it is used for the rehabilitation of adults living with neurological conditions worldwide.

**Objective:**

We aimed to provide a comprehensive overview of 3D printing in neurorehabilitation and precisely explore how it is used to improve motor recovery for adults with neurological conditions living in higher- and lower-middle–income countries.

**Methods:**

We conducted a scoping review following the Joanna Briggs Institute guidelines. After searching 3 databases (MEDLINE, Web of Science, and Nursing and Allied Health Premium), 2 independent reviewers screened and selected English-language studies involving adults (≥18 years) published between 2019 and 2024 to capture the most recent advancements in this field. We extracted relevant information on neurological conditions, motor recovery outcomes, and types of 3D printing and offered a comparative analysis of 3D printing in physical neurorehabilitation from the perspective of national income levels using a modified Joanna Briggs Institute extraction form. We synthesized the findings narratively with tabular support.

**Results:**

After screening 2752 titles and abstracts and 103 (3.7%) full texts, we included 13 (0.5%) studies based on our inclusion criteria. All included studies were conducted in upper-middle–income or high-income countries, and most studies (9/13, 69.2%) focused on stroke, followed by spinal cord injury (2/13, 15.4%), Parkinson disease (1/13, 7.7%), and central nerve disease (1/13, 7.7%). The 3D-printed rehabilitation tools included orthotics (7/13, 53.8% for the upper extremities [UEs]; 3/13, 23.1% for the lower extremities [LEs]), an exoskeleton (1/13, 7.7%; UEs), a modular assistive hand device (1/13, 7.7%; UEs), and an insole (1/13, 7.7%; LEs). In total, 69.2% (9/13) of the studies targeted UE rehabilitation, measured using the Action Research Arm Test, active range of motion, the box and block test, the Fugl-Meyer Assessment, the Modified Ashworth Scale, the manual function test, range of motion, and the Toronto Rehabilitation Institute Hand Function Test, and 30.8% (4/13) targeted LE rehabilitation, measured using the 10-m walk test, anteroposterior ground reaction force analysis, the Barthel index, the Tinetti scale, the RehaWatch system, and the GaitWatch system.

**Conclusions:**

Used as a rehabilitation tool, 3D printing technology has demonstrated significant potential in improving upper and lower motor recovery for people with certain neurological conditions in high-middle–income countries. Future research should explore the implementation feasibility and effectiveness of these technologies across different neurological conditions and income settings, particularly in low- and lower-middle–income countries.

## Introduction

### Background

Neurological conditions encompass a broad spectrum of central and peripheral conditions caused by damage to the brain, spinal cord, or peripheral nerves that interfere with brain development or result in impaired motor functions [1]. According to a 2021 report published in *The Lancet Neurology* by the World Health Organization (WHO), over 3 billion people in the world are living with a neurological condition [2,3]. Neurological conditions such as stroke, traumatic brain injury, spinal cord injury, cerebral palsy, Parkinson disease, and cognitive disorders are some of the leading causes of illness and disability worldwide [2,4,5]. Disability-adjusted life years, which measure the overall burden of disease, including disability, sickness, and premature death due to neurological conditions, have increased by 18% since 1990 [1-3]. Moreover, care disparities persist across demographics. Higher-income countries possess greater access to specialized care, with 70 times more health professionals specializing in neurological conditions per 100,000 individuals compared to lower-middle–income countries [1]. In the United States alone, the financial and public health burden of neurological conditions is estimated to cost US $1.7 trillion annually [1]. Given the global prevalence of neurological conditions, and with over 80% of neurological-related deaths and health losses occurring in low- and middle-income countries (LMICs), there is a need for effective treatment strategies to improve patient health outcomes and reduce associated burdens worldwide [1-7]. Neurorehabilitation is an interdisciplinary clinical process designed to assess disabilities and provide rehabilitation interventions to enhance functions affected by damage to the nervous system [8,9]. The WHO defines neurorehabilitation as enabling individuals to acquire the knowledge, skills, and resources necessary to achieve optimal physical, psychological, social, and economic functioning [2,3,8]. Many interventions, including assistive devices, therapeutic exercises, and cognitive behavioral therapies, are used in the provision of rehabilitation services for individuals with neurological conditions [5,10,11]. For motor recovery, one promising rehabilitation tool is the use of 3D printing technology.

This is an emerging technology that creates 3D objects out of melting materials into thin layers printed layer by layer based on computer-programmed designs [12]. A range of materials can be used, including nylon, biodegradable plastic filament, polylactic acid, and acrylonitrile butadiene styrene plastic [12,13]. Worldwide, 3D printing has the potential to improve access to rehabilitation devices in lower-middle–income countries where traditional manufacturing processes can be both costly and time-consuming [14,15]. Currently, in health care, 3D printing has been widely adopted beyond rehabilitation, including applications in surgical planning, dental restorations, and the production of anatomical models for medical education and preoperative simulation [16]. In the rehabilitation field, 3D printing is being used to produce a variety of highly customized, patient-specific devices, such as orthotics, prosthetics, and assistive tools [6,12,14]. Many of these designs are low cost, customizable, and easy to assemble, making them well suited for resource-limited settings [12,15]. Specific to neurorehabilitation, 3D printing technologies have gained increasing attention for their potential to deliver personalized, cost-effective, and accessible therapeutic solutions [17]. Despite growing interest, there remains limited synthesis of how these technologies are being applied worldwide, particularly in relation to their use in supporting motor recovery in adults living with neurological conditions. As the scope of neurorehabilitation extends across multiple professions (ie, physiotherapy, occupational therapy, audiology, speech-language pathology, social work, and nursing), focusing on motor recovery specific to physiotherapy or physical rehabilitation is a first step toward understanding 3D printing use in neurorehabilitation.

### Research Objectives

To address this gap, the objective of our scoping review was to provide a comprehensive overview of how 3D printing technologies have recently been used in neurorehabilitation for adults. We aimed to document and compare their applications across high-income, upper-middle–income, lower-middle–income, and low-income countries and identify the types of assessments used to evaluate motor recovery outcomes when using 3D-printed rehabilitation tools, including standardized clinical scales, functional performance tests, and biomechanical measurements. To our knowledge, this study is the first to offer a comparative analysis of 3D printing in physical neurorehabilitation from the perspective of national income levels.

## Methods

We conducted this scoping review in accordance with the Joanna Briggs Institute (JBI) methodology for scoping reviews [17] and aimed to answer the following question: how is 3D printing used to improve motor recovery for adults with neurological conditions in higher- and lower-middle–income countries?

### Population, Concept, and Context Framework

We used the population, concept, and context framework as recommended by the JBI guidelines [17]. We included studies involving adult participants (18 years and older) with neurological conditions who received rehabilitation interventions aimed at improving or restoring motor function. Participants under 18 years of age were excluded as pediatric populations have different developmental trajectories and rehabilitation needs, which fall outside the scope of this review [18]. Eligible conditions included acquired brain injuries, neurodegenerative disorders, and traumatic brain or spinal cord injuries [19].

The concept for this review focused on the application of 3D printing technology in improving motor recovery for adults with neurological conditions. Studies exploring the use of 3D printing rehabilitation interventions or 3D-printed tools to enhance motor function in adults within multiple rehabilitation settings were considered. Rehabilitation interventions included physiotherapy or physical rehabilitation strategies designed to enhance motor abilities. Physiotherapy referred to evidence-based treatments delivered by trained professionals to restore movement and function, often through targeted exercises, manual techniques, and assistive devices. Physical rehabilitation strategies more broadly included any structured interventions aimed at improving motor control, strength, or mobility, particularly after neurological injury. Settings included clinical environments (eg, hospitals, rehabilitation centers, and outpatient clinics), home-based rehabilitation programs, and laboratory-based rehabilitation programs. Studies that focused on 3D printing for surgical planning, medical pharmaceutics, bioengineering, or any interventions that did not report clinical or functional motor recovery outcomes (ie, purely descriptive studies) were excluded as they did not reflect the rehabilitation objective of this review [16].

This scoping review considered studies conducted in contexts of higher-income, upper-middle–income, lower-middle–income, and low-income countries as defined by the World Bank based on gross national income per capita for 2023 (Multimedia Appendix 1) [20]. The geographical and socioeconomic context was explored in terms of access to 3D printing technologies as well as how this impacts the effectiveness of these interventions. No limitations were placed on the gender, ethnicity, or religion of the participants included in the studies.

### Developing a Search Strategy

We developed a rigorous search strategy in 3 steps. First, we conducted a preliminary search of MEDLINE (PubMed) to identify articles on the topic and define main keywords. In this preliminary search, we used the keywords “3D printing” and “rehabilitation” to capture as many articles on the topic as possible, thus providing insights into existing literature. We then reviewed keywords contained in titles and abstracts and the indexing terms used to describe potentially included articles (reviewing keywords and indexing terms from relevant titles and abstracts), which was then used to refine our search strategy. Second, we developed a refined search strategy incorporating additional keywords: “physiotherapy,” “physical therapy,” or “physical rehabilitation” (Multimedia Appendix 2). This refined search strategy incorporated both MeSH (Medical Subject Headings) terms and text word searches in MEDLINE (PubMed) and was adapted using equivalent text word strategies in 2 other databases (Web of Science and Nursing and Allied Health Premium). In this step, a filter for English-language publications was applied across all databases to maintain consistency (Multimedia Appendix 2). Third, the reference lists of all articles included in the review were manually screened for additional relevant papers. This step aimed to capture any pertinent studies that may not have been identified through the database searches.

### Study Screening and Evidence Selection

Following the 3-database search, we collated all identified studies and used Mendeley Reference Manager (version 2.51.0; Elsevier) and Covidence (Veritas Health Innovation) for citation management and removal of duplicates. Titles and abstracts were screened by 2 independent reviewers (SA and ZL) for assessment against the inclusion and exclusion criteria. We screened studies published from 2019 onward, with the final search completed on November 21, 2024, to capture the most recent advancements in 3D printing technologies for neurorehabilitation. We included a variety of study designs (ie, randomized controlled trials [RCTs], nonrandomized experimental studies, pilot and feasibility studies, case studies, and systematic reviews) in our search to capture both the effectiveness of the interventions and the practical considerations surrounding their implementation. RCTs and non-RCTs were included to assess potential causal relationships between 3D printing interventions and motor recovery outcomes [21]. Pilot and feasibility studies were eligible if they examined the usability, acceptability, or logistical feasibility of 3D-printed devices in rehabilitation contexts [22]. Case studies were included if they provided detailed examples of individualized application [23]. Systematic reviews were considered if they synthesized primary research aligned with our inclusion criteria [24]. Opinion pieces, editorials, commentaries, and protocols without full-text availability were excluded as they did not contribute to the empirical evidence required for conclusions. When the inclusion criteria were met at the title and abstract screening stage, the full texts were screened through the same 2-reviewer screening method. Any disagreements were resolved through consensus with a third reviewer (JON) when necessary.

### Data Extraction

We extracted relevant data from the included full-text studies. We used a data extraction form that was developed based on the JBI guidelines (Multimedia Appendix 3) to ensure completeness of data extraction. The extraction form included (1) general study characteristics, including authors, title, year of publication, date of data collection, study type, country, and study aim; (2) population details, such as sample size, age, gender, and the health condition being addressed; (3) intervention details, including setting (eg, clinical, home based, or laboratory) and duration of the intervention, type of 3D-printed technology used (eg, orthosis or insole), stiffness and type of polymer, neurological condition (eg, stroke or Parkinson disease), and motor area focus (upper extremities [UEs] or lower extremities [LEs]); and (4) outcomes, including the types of assessments used to evaluate motor recovery, such as standardized clinical scales, functional performance tests, and biomechanical measurements.

### Data Analysis and Interpretation

We analyzed the data using descriptive qualitative content analysis and synthesized the extracted data from a geographical perspective. Data were systematically mapped based on the objectives of our scoping review to capture study characteristics, (authors, title, and publication year), the type of 3D-printing technology used, the associated neurological condition and rehabilitation goal, outcome measures, and country of implementation. A comparative approach was applied to assess differences between high-income and lower-middle–income countries, documenting disparities in access (as reported through the availability of 3D printing technology, affordability considerations, descriptions of infrastructure, and integration into clinical practice) and effectiveness. Findings were synthesized into thematic categories, providing a comprehensive overview of how 3D-printed technologies have been used to support motor recovery in different health care contexts.

## Results

### Overview

**Figure 1.**
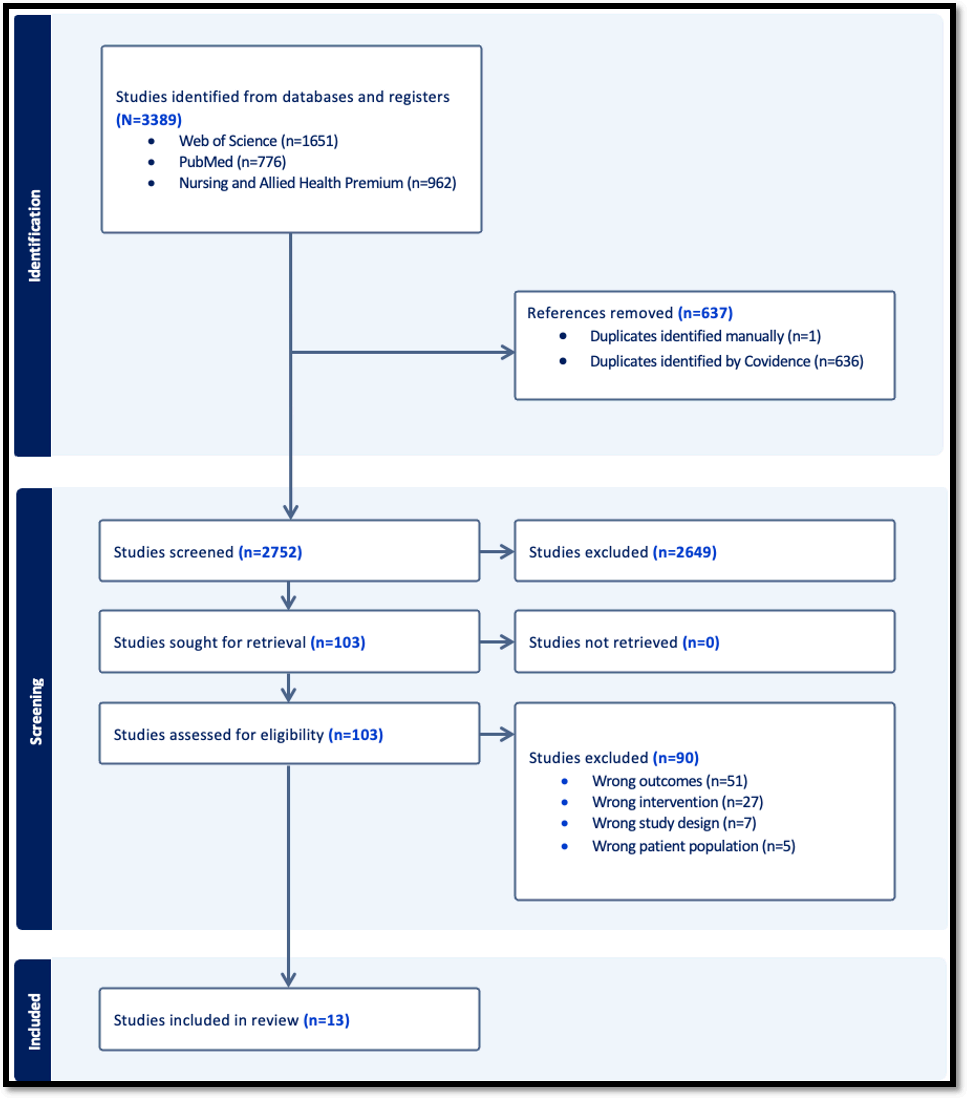
PRISMA (Preferred Reporting Items for Systematic Reviews and Meta-Analyses) flowchart showing the selection of studies for inclusion in the scoping review.

A total of 2752 titles and abstracts were screened. Of these 2752 articles, after removing 637 (23.1%) duplicates, 103 (3.7%) full-text articles were assessed for eligibility, and 90 (3.3%) were excluded (Figure 1). We included 13 studies [25-37], with sample sizes ranging from single case reports (N=1 individual) to small trials (N=31 individuals) (Table 1). The reported participant ages ranged from 23 to 83 years, aligning with the inclusion criterion of adult participants (18 years and older).

**Table 1.** Technologies used in neurological rehabilitation for upper- and lower-extremity impairments, including the country in which the technology was implemented, the associated neurological condition, the type of technology, 3D printing materials and processes, the outcome summary, and the outcome measures used to assess effectiveness.

Study and country	Neurological condition	Assistive device	3D printing details (device; stiffness and type of polymer) and process	Outcome summary	Outcome measures
Upper-extremity impairments					
Chen et al [25], 2022, China	Stroke	Hand splint	PLA^a^; 3D printing method and process not specified	Improved grip and lateral pinch strength; no significant change in palmar pinch and gross movement	ARAT^b^ and pinch force tests^c^
Demeco et al [26], 2023, country not specified	Stroke	Hand splint	Not specified	Improved upper-limb ROM^d^, hand dexterity, and pinch strength (palmar and lateral); reduced spasticity	ROM, BBT^e^, pinch force tests, FMA^f^, and MAS^g^
Dudley et al [27], 2021, United States	Stroke	Exoskeleton	PLA+1% copper additive (PLACTIVE); FDM^h^ (Ultimaker 2+ Extended); infill: 35%‐40%; layer height: 0.15‐0.25 mm; bed: 50 °C; shell: 0.8 mm; print speed: 60‐100 mm/s	Improved finger flexion and extension, grasp function, and hand dexterity	FMA and BBT
Huber et al [28], 2023, United States	Stroke	Hand splint	Variable-stiffness TPU^i^ powder; SLS^j^ 3D printing; printer and parameters not reported	Improved hand dexterity and pincer force; no significant change in pincer aperture	Pincer force test, pincer aperture test, and BBT
Kuo et al [29], 2024, China	Neurological central nerve injury	Modular assistive hand technology device	TPU filament (1.75 mm); FDM (Original Prusa i3 MK3S+); CAD^k^ designed (SolidWorks, DesignSpark Mechanical, and Meshmixer)	Improved shoulder AROM^l^ and functional hand tasks; no significant change in hand dexterity or grip strength	AROM, BBT, Jamar dynamometer, and functional hand tasks
Yeh et al [30], 2023, China	Spinal cord injury	Hand splint	Material not specified; FDM (FlashForge Finder); layer height: 0.2 mm; infill: 15%; speed: 60‐80 mm/s; print temperature: 200 °C; nonprinted components: silicone finger cots; acrylic linkages (3 mm) laser cut	Improved pinch force and hand dexterity	Pinch force test and BBT
Toth et al [31], 2020, Hungary	Stroke	Hand splint	PA^m^ 2200 (nylon) framework; TPU sheets; nitinol SMA^n^+Kapton heating elements; SLS (EOS Formiga P 110); 110 μm layers; FDM; integrated heating elements	Improved pencil holding, handle and tool grasp, vertical handle grasp, cup holding, door handle operation, and device assembly; no significant improvement in eraser holding	MFT^o^
Yang et al [32], 2021, China	Stroke	Hand splint	ABS^p^ filament; FDM (UP Box); layer thickness: 0.2 mm; infill: 20%; nozzle temperature: 230 °C; print speed: 60 mm/s	Improved upper-extremity motor function; reduced spasticity	MAS and FMA
Yoo et al [33], 2019, South Korea	Spinal cord injury	Hand splint	PLA; FDM (Moment 2); CAD designed (SolidWorks); postprint heat reshaping; nonprinted components: self-adhesive padding, Velcro, and nylon thread	Improved grasp, lift, and object manipulation	TRI-HFT^q^
Lower-extremity impairments					
Brognara et al [34], 2020, Spain	Parkinson disease	Insole	Material not specified; 3D foot scan (3D Sense); CAD designed (Rhinoceros 3D); FDM (Delta WASP 4070); custom insole with 2 blunted cones (plantar stimulation)	Improved balance; no significant change in daily activities	Barthel index and Tinetti scale
Hsu et al [35], 2020, China	Stroke	Ankle-foot orthoses	Nylon (4611); 3D scan (Structure Sensor; OBJ^r^→STL^s^); FDM (Ultimaker); approximately 3-mm wall thickness	Improved gait parameters; no significant change in walking speed	RehaWatch system and 10MWT^t^
Kwon et al [36], 2019, China	Stroke	Ankle-foot orthoses	TPU filament; VeroBlackPlus (rigid components); FDM (Cubicon Single Plus); PolyJet (Objet 30 Prime)	Improved paretic propulsion; no significant change in walking speed	Anteroposterior ground reaction force analysis and 10MWT
Liu et al [37], 2019, South Korea	Stroke	Ankle-foot orthoses	PA-12; 3D scan (EinScan-Pro); model modification (Geomagic Studio); MJF^u^	Increased gait velocity and stride length; no significant changes in cadence and step symmetry	GaitWatch system

a PLA: polylactic acid.

b ARAT: Action Research Arm Test.

c “Pinch force test” is used as a standardized term across studies measuring pinch strength except for the study by Huber et al [28], which specifically used “pincer force test” based on a defined protocol.

d ROM: range of motion.

e BBT: box and block test.

f FMA: Fugl-Meyer Assessment.

g MAS: Modified Ashworth Scale.

h FDM: fused deposition modeling.

i TPU: thermoplastic polyurethane.

j SLS: selective laser sintering.

k CAD: computer-aided design.

l AROM: active ROM.

m PA: polyamide.

n SMA: shape-memory alloy.

o MFT: manual function test.

p ABS: acrylonitrile butadiene styrene.

q TRI-HFT: Toronto Rehabilitation Institute Hand Function Test.

r OBJ: Wavefront Technologies file format.

s STL: stereolithography.

t 10MWT: 10-m walk test.

u MJF: multijet fusion.

### Overview of 3D-Printed Rehabilitation Tools Used in Neurorehabilitation per Country

**Figure 2.**
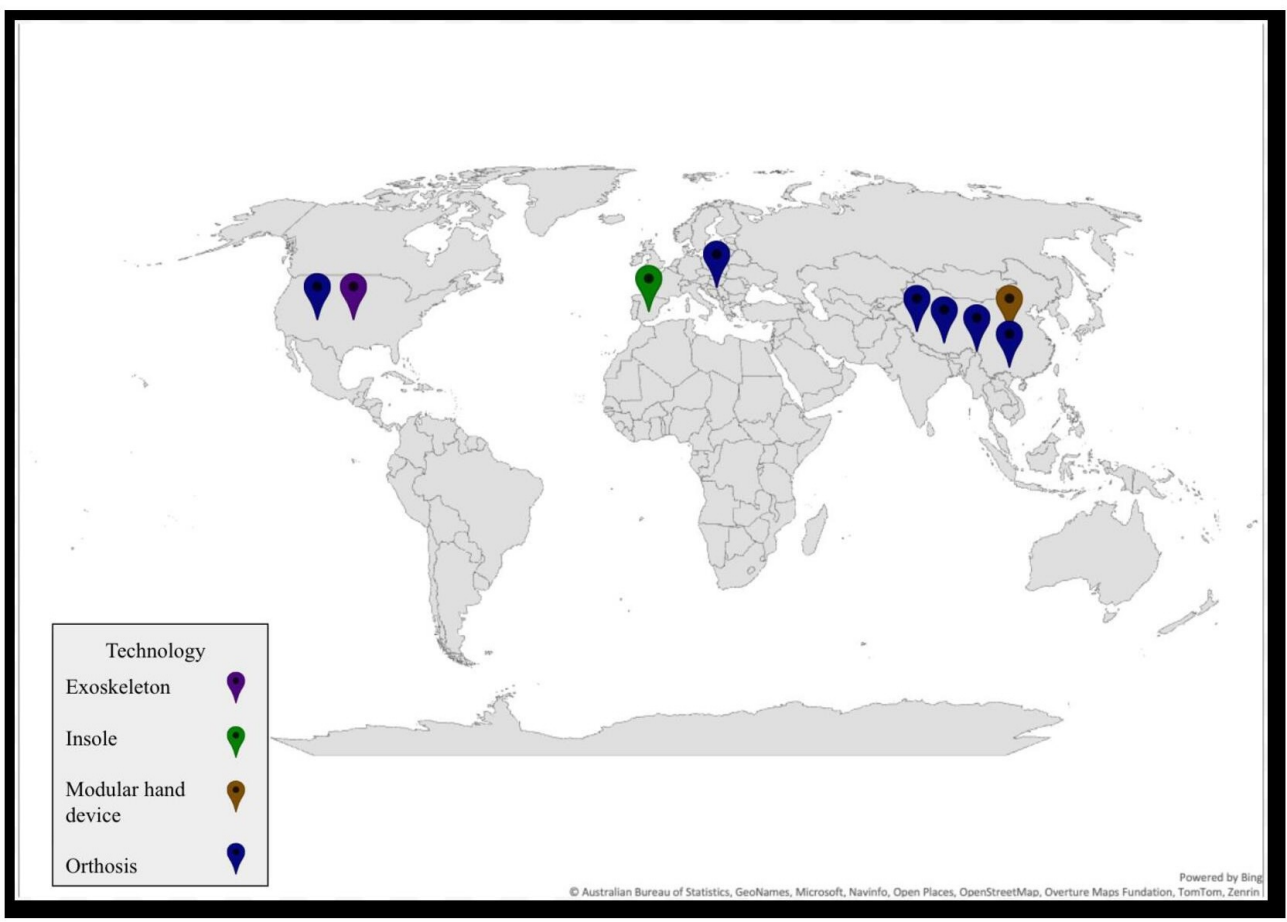
Geographical distribution of 3D-printed rehabilitation tools by country and technology type. Map created using Microsoft Excel and Google Drawings.

All included studies [25-37] were conducted in upper-middle–income or high-income countries (ie, China, South Korea, the United States, Spain, and Hungary; Figure 2). While 4 different types of neurorehabilitation tools were designed using 3D printing—orthotics (10/13, 76.9%) [25,26,28,30-33,35-37], an exoskeleton (1/13, 7.7%) [27], a modular assistive hand technology device (1/13, 7.7%) [29], and an insole (1/13, 7.7%) [34] (Multimedia Appendix 4)—most included studies (8/13, 61.5%) primarily focused on orthoses for stroke rehabilitation [25,26,28,30-32,35-37].

In total, 69.2% (9/13) of the included studies focused on rehabilitation of UE function, and 30.8% (4/13) focused on LE rehabilitation [25,27-33]. For UE rehabilitation, 5 of the studies used 3D printing technology to design hand splints for stroke-related impairments [25,26,28,31,32], 2 of the studies focused on hand splints for individuals with spinal cord injury [30,33], 1 study included a 3D-printed modular assistive hand device for patients with central nerve injuries [29], and 1 study investigated the use of a 3D-printed upper-limb exoskeleton to support hand rehabilitation for individuals after stroke [27]. For LE rehabilitation, 3 of the studies explored the use of 3D-printed ankle-foot orthoses for gait impairment rehabilitation after stroke [35-37], and 1 study used a 3D-printed insole for people living with Parkinson disease [34].

### Types of Assessments

Across studies, outcome measures were predominantly used in pretest-posttest designs to assess changes in motor performance before and after the application of 3D-printed rehabilitation tools. The most frequently used standardized outcome measures for UEs were the pinch force test (4/13, 30.8%) [25,26,28,30] and the box and block test (5/13, 38.5%) [26-30]. For LEs, a commonly used outcome measure was the 10-m walk test (10MWT; 2/13, 15.4%) [35,36].

## Discussion

### Principal Findings

To our knowledge, this scoping review is the first to explore the application of 3D printing rehabilitation interventions for adults with neurological conditions from a geographical perspective. We selected a geographical perspective and the World Bank national income classification to reflect the socioeconomic and structural determinants of adoption that may influence whether 3D-printed neurorehabilitation devices can be implemented across international health systems. Our geographical analysis showed that rehabilitation research on 3D printing technologies is primarily concentrated in higher- and upper-middle–income countries (ie, China, the United States, South Korea, Spain, and Hungary). This distribution reflects broader economic trends and access to infrastructure and materials. In contrast to this technological growth in higher-income countries, rehabilitation-specific data remain limited in lower- and lower-middle–income countries. No studies were identified in our review from lower-middle– or low-income countries, highlighting the current gap in implementation within resource-constrained health systems.

As outlined in the WHO *Global strategy on digital health 2020‐2025*, such disparities reflect systemic barriers in low-resource settings, including underdeveloped infrastructure, limited digital capacity, and inadequate financing models that constrain the adoption of digital health technologies [38]. Potential barriers also include the cost of medical-grade 3D printers, which can be substantial depending on material capabilities; infrastructure limitations such as unreliable electricity, insufficient internet connectivity, and shortages of trained personnel [14,39]; sociocultural factors such as potential stigma associated with 3D-printed assistive technologies; and if rehabilitation is not widely used in some regions, this may further affect the uptake of 3D-printed devices for rehabilitation [14,40]. The technical compatibility of 3D printing in lower-middle– or low-income countries may also be limited by gaps in the broader fabrication ecosystem required for clinical device production. This includes unreliable access to printing materials (eg, filament or resin), supplementary assistive device components (eg, straps or Velcro and padding or foam), and the software and digital infrastructure needed for the design process [12,14,40]. Even when 3D printers are available, limited capacity for postprocessing (eg, curing and edge finishing), routine maintenance (eg, calibration and replacement of wear parts), and training of clinicians may hinder sustained implementation in resource-constrained settings [12,14,40]. While barriers must be considered, investigating the use of emerging technologies in LMIC settings is crucial to understanding adoption, adaptation, and implementation in various and diverse contexts. The feasibility and long-term sustainability of 3D printing in low-resource settings remain critical areas for future investigation [14,40].

### 3D Printing Technology in Neurorehabilitation

Integrating 3D printing technologies into neurorehabilitation represents a paradigm shift in delivering innovative, personalized, clinically relevant interventions for individuals with neurological conditions. Our scoping review synthesized evidence on the application of novel technology, including 3D-printed orthotics, exoskeletons, modular assistive hand devices, and insoles, supporting motor rehabilitation for individuals who have experienced a stroke or spinal cord injury or been diagnosed with Parkinson disease or central nerve injury. We recognize that these are not representative of all neurological conditions, and we consider that our findings suggest a potential shift characterized by the ability to use emerging technologies such as 3D printing to rapidly design and produce patient-specific rehabilitation tools tailored to individual needs and functional impairments [12]. Among the technologies reviewed, orthotic devices emerged as the most widely studied, primarily addressing lower motor impairments for persons who experienced a stroke. This focus is particularly important in both upper-middle– and lower-income countries as stroke remains a leading cause of morbidity and mortality worldwide. Gait impairments after stroke are extensively researched and prevalent in neurological rehabilitation, so investigating cost- and resource-effective tools tailored to these impairments is imperative [41]. Unlike conventional rehabilitation tools, 3D-printed solutions may offer greater flexibility and adaptive adjustments based on the patient, faster production timelines, and affordability [12,14]. Therefore, 3D-printed rehabilitation tools may improve fit, comfort, and usability to enhance motor recovery outcomes and support greater independence in daily activities. Their application across clinical and home-based settings could further broaden access to care, particularly in LMICs. Given this emphasis and the growing potential of 3D printing in neurorehabilitation, it is essential that future research not only continue to refine orthotic solutions but also explore a broader range of technologies and document their effectiveness within neurorehabilitation contexts.

Our findings offer a comprehensive overview of measurements used to evaluate motor recovery outcomes (ie, standardized clinical, functional, and biomechanical measurements). Gold-standard rehabilitation assessments are those that are widely validated, clinically reliable, and consistently used across diverse populations to measure functional outcomes with high sensitivity and specificity. Results from our scoping review identified 4 gold-standard outcome measures being used for assessing the impact of 3D printing technologies on UE rehabilitation: the Fugl-Meyer Assessment (FMA), the Action Research Arm Test (ARAT), the Jamar dynamometer, and the Barthel index. For LE rehabilitation, the 10MWT was the only gold-standard outcome measure identified. The frequent use of the FMA across our included studies reflects its widespread acceptance as an outcome measure, particularly for people after stroke. Its strong psychometric properties, such as high interrater reliability, responsiveness to change, and internal consistency, make it a clinically relevant tool for capturing motor function in the UEs [42-44]. Within the context of 3D printing interventions, the FMA’s emphasis on voluntary movement patterns, coordination, and reflex activity aligns well with the types of impairments that these technologies aim to address [42-44]. Its application in studies examining customized orthoses and exoskeletons suggests that researchers prioritize outcome measures that can accommodate the complexity of stroke-related motor deficits and individualized recovery trajectories [42-45].

The Jamar dynamometer was used in 7.7% (1/13) of the studies [29] and serves as a recognized gold-standard tool for objectively quantifying grip strength. In neurorehabilitation, grip strength is a meaningful proxy for upper-limb recovery and is often correlated with broader indicators of functional independence and quality of life in individuals with neurological impairments [46,47]. The Jamar dynamometer is particularly important for evaluating 3D printing interventions as it can provide precise, quantifiable feedback on how assistive devices, such as modular assistive hand devices, can enhance grip function and hand strength for adults with neurological conditions [46,47]. These objective data strengthen the clinical evidence supporting the functional efficacy of 3D-printed devices and enable more informed design refinements for 3D-printed technologies. In resource-limited settings, where comprehensive neurological assessments may not be feasible, dynamometers can offer practical and scalable solutions for tracking fine motor outcomes in response to 3D printing interventions [48].

The Barthel index was used in 7.7% (1/13) of the studies [34] as a gold-standard tool and captures independence in essential activities of daily living, including feeding, grooming, bathing, mobility, and toileting [43,49]. Its relevance lies in its ability to assess the effectiveness of 3D-printed insoles by monitoring changes in self-care and mobility over time, thereby capturing the progressive nature of degenerative neurological conditions such as Parkinson disease [50]. Its ease of administration, low cost, and global familiarity make it especially well suited for integration into 3D printing rehabilitation protocols in settings with limited infrastructure or personnel [49].

The ARAT was reported in 7.7% (1/13) of the studies [25] as a gold-standard measure to evaluate upper-limb performance across 4 domains: grip, grasp, pinch, and gross movement [51]. The ARAT has widely excellent interrater reliability, high test-retest reliability, and good construct validity, particularly for people after stroke [51]. By assessing both fine and gross components of upper-limb function, it is well suited for evaluating 3D-printed devices targeting grasp and release, dexterity, and functional hand use [25,51].

The 10MWT was used in 15.4% (2/13) of the studies [35,36] as a gold-standard measure of short-distance walking performance. Its relevance to 3D-printed ankle-foot orthoses lies in its ability to quantify clinically meaningful changes in gait capacity through walking speed, a core functional indicator of mobility and community ambulation after neurological injury [52,53]. The 10MWT is particularly well suited for integration into 3D printing rehabilitation protocols in resource-limited settings because it is rapid to administer and requires minimal equipment and training for clinicians and patients [52,53].

The systematic use of gold-standard rehabilitation assessments within studies investigating 3D printing interventions is particularly significant as they provide an important framework for objectively evaluating the efficacy of these technologies, ensuring that observed improvements in motor function are both measurable and clinically meaningful across various neurological populations. Therefore, by aligning 3D printing research with gold-standard tools, clinicians and researchers can ensure more accurate, comparable, and clinically meaningful assessments of motor recovery and functional gains.

### Strengths and Limitations

A strength of this scoping review lies in its rigorous methodology, which included a comprehensive search strategy, clear inclusion criteria, and a structured data extraction form. By synthesizing evidence across diverse neurological conditions and outcome measures, this review provides a detailed understanding of how 3D-printed technologies contribute to motor recovery.

Although previous research has demonstrated improvements in motor recovery outcomes through 3D-printed rehabilitation technologies, several limitations remain. Studies included in this review had small sample sizes and heterogeneous intervention protocols, limiting the ability to draw generalizable conclusions. Additionally, the search strategy may have inadvertently excluded relevant studies by not expanding the keywords to include other discipline-specific terminology (eg, “occupational therapy,” “audiology,” and “nursing”). Not indexing these terms potentially narrowed the scope of the findings. Moreover, restricting inclusion to English-language publications may have introduced a language bias and limited the generalizability of the results.

All eligible studies were conducted in high- or middle-income countries, which limited comparisons across income groups and may have contributed to heterogeneity as the effectiveness and feasibility of 3D-printed neurorehabilitation devices may differ in infrastructure-constrained settings. Printer type, printing materials, and fabrication processes were inconsistently reported across the included studies. We reported available details descriptively (Table 1) but were unable to synthesize fabrication methods across studies or provide implementation guidance.

### Conclusions

This scoping review highlights the growing interest in 3D-printed rehabilitation technologies within neurorehabilitation, particularly in the development of orthoses for motor recovery [12,14]. While preliminary findings suggest that 3D printing may offer a flexible and cost-effective means of delivering personalized interventions, the current evidence base remains limited by small sample sizes and heterogeneous study designs [14]. To strengthen the field, future research should prioritize larger and more diverse populations; standardized protocols; and consistent use of validated, clinically meaningful outcome measures [54]. These efforts are essential to better assess the functional impact and real-world applicability of 3D-printed technologies in neurorehabilitation [54].

## Supplementary material

10.2196/81782 Multimedia Appendix 1 Classification of countries by income level (2023).

10.2196/81782 Multimedia Appendix 2 Search query.

10.2196/81782 Multimedia Appendix 3 Modified Joanna Briggs Institute data extraction form.

10.2196/81782 Multimedia Appendix 4 Distribution of 3D-printed rehabilitation tools by health condition and motor areas.

10.2196/81782 Checklist 1 PRISMA-ScR checklist.

## Abbreviations

10MWT: 10-m walk test
ARAT: Action Research Arm Test
FMA: Fugl-Meyer Assessment
JBI: Joanna Briggs Institute
LE: lower extremity
LMIC: low- and middle-income country
MeSH: Medical Subject Headings
RCT: randomized controlled trial
UE: upper extremity
WHO: World Health Organization

## References

[1] Steinmetz J, Seeher K, Schiess N et al (2024). Global, regional, and national burden of disorders affecting the nervous system, 1990-2021: a systematic analysis for the Global Burden of Disease Study 2021. Lancet Neurol.

[2] WHO Media Team (2024). Over 1 in 3 people affected by neurological conditions, the leading cause of illness and disability worldwide. World Health Organization.

[3] The Lancet Neurology (2023). Sex, gender, and the cost of neurological disorders. Lancet Neurol.

[4] Abuín-Porras V, Martinez-Perez C, Romero-Morales C (2022). Citation network study on the use of new technologies in neurorehabilitation. Int J Environ Res Public Health.

[5] Tamburin S, Smania N, Saltuari L, Hoemberg V, Sandrini G (2019). Editorial: New advances in neurorehabilitation. Front Neurol.

[6] Oña ED, Cano-de la Cuerda R, Sánchez-Herrera P, Balaguer C, Jardón A (2018). A review of robotics in neurorehabilitation: towards an automated process for upper limb. J Healthc Eng.

[7] Dinon T, Caimmi M, Chiavenna A (2018). DUALarm: an open-source and 3D-printable device for upper limb neurorehabilitation. J Rehabil Assist Technol Eng.

[8] Burke S, McGettrick G, Foley K, Manikandan M, Barry S (2020). The 2019 neuro-rehabilitation implementation framework in Ireland: challenges for implementation and the implications for people with brain injuries. Health Policy.

[9] Iosa M, Verrelli CM, Gentile AE, Ruggieri M, Polizzi A (2022). Gaming technology for pediatric neurorehabilitation: a systematic review. Front Pediatr.

[10] Khan F, Amatya B, Galea MP, Gonzenbach R, Kesselring J (2017). Neurorehabilitation: applied neuroplasticity. J Neurol.

[11] Schneider WN, Wong TM (2011). Cognitive and behavioral disorders in neurorehabilitation. Continuum (Minneap Minn).

[12] Ishengoma FR, Mtaho AB (2014). 3D printing: developing countries perspectives. Int J Comput Appl.

[13] Shahrubudin N, Lee TC, Ramlan R (2019). An overview on 3D printing technology: technological, materials, and applications. Procedia Manuf.

[14] Abbady HE, Klinkenberg ET, de Moel L (2022). 3D-printed prostheses in developing countries: a systematic review. Prosthet Orthot Int.

[15] Dally C, Johnson D, Canon M, Ritter S, Mehta K Characteristics of a 3D-printed prosthetic hand for use in developing countries.

[16] Dodziuk H (2016). Applications of 3D printing in healthcare. Kardiochir Torakochirurgia Pol.

[17] Aromataris E, Lockwood C, Porritt K, Pilla B, Jordan Z (2024). JBI Manual for Evidence Synthesis.

[18] Kaufman JN, Lahey S, Slomine BS (2017). Pediatric rehabilitation psychology: rehabilitating a moving target. Rehabil Psychol.

[19] Carr JH, Shepherd RB (2010). Neurological Rehabilitation: Optimizing Motor Performance.

[20] Roser M, Rohenkohl B, Arriagada P, Hasell J, Ritchie H, Ortiz-Ospina E (2025). World Bank income groups. Our World in Data.

[21] Verde PE, Ohmann C (2015). Combining randomized and non-randomized evidence in clinical research: a review of methods and applications. Res Synth Methods.

[22] Lawson DO, Mellor K, Eddy S (2022). Pilot and feasibility studies in rehabilitation research: a review and educational primer for the physiatrist researcher. Am J Phys Med Rehabil.

[23] Rowley J (2002). Using case studies in research. Manag Res News.

[24] Whitlock EP, Lin JS, Chou R, Shekelle P, Robinson KA (2008). Using existing systematic reviews in complex systematic reviews. Ann Intern Med.

[25] Chen ZH, Yang YL, Lin KW, Sun PC, Chen CS (2022). Functional assessment of 3D-printed multifunction assistive hand device for chronic stroke patients. IEEE Trans Neural Syst Rehabil Eng.

[26] Demeco A, Foresti R, Frizziero A (2023). The upper limb orthosis in the rehabilitation of stroke patients: the role of 3D printing. Bioengineering (Basel).

[27] Dudley DR, Knarr BA, Siu KC, Peck J, Ricks B, Zuniga JM (2021). Testing of a 3D printed hand exoskeleton for an individual with stroke: a case study. Disabil Rehabil Assist Technol.

[28] Huber J, Slone S, Bazrgari B (2023). An evaluation of 3D printable elastics for post stroke dynamic hand bracing: a pilot study. Assist Technol.

[29] Kuo FL, Wu YS, Kuo TY, Lee YS, Huang SW, Lee HC (2024). Effects of 3D-printed assistive device on daily life function in patients with neurological impairment: a pilot study. Disabil Rehabil Assist Technol.

[30] Yeh PC, Chen CH, Chen CS (2023). Using a 3D-printed hand orthosis to improve three-jaw chuck hand function in individuals with cervical spinal cord injury: a feasibility study. IEEE Trans Neural Syst Rehabil Eng.

[31] Toth L, Schiffer A, Nyitrai M, Pentek A, Told R, Maroti P (2020). Developing an anti-spastic orthosis for daily home-use of stroke patients using smart memory alloys and 3D printing technologies. Mater Des.

[32] Yang YS, Tseng CH, Fang WC, Han IW, Huang SC (2021). Effectiveness of a new 3D-printed dynamic hand-wrist splint on hand motor function and spasticity in chronic stroke patients. J Clin Med.

[33] Yoo HJ, Lee S, Kim J, Park C, Lee B (2019). Development of 3D-printed myoelectric hand orthosis for patients with spinal cord injury. J Neuroeng Rehabil.

[34] Brognara L, Navarro-Flores E, Iachemet L, Serra-Catalá N, Cauli O (2020). Beneficial effect of foot plantar stimulation in gait parameters in individuals with Parkinson’s disease. Brain Sci.

[35] Hsu CY, Wu CM, Huang CC, Shie HH, Tsai YS (2022). Feasibility and potential effects of robot-assisted passive range of motion training in combination with conventional rehabilitation on hand function in patients with chronic stroke. J Rehabil Med.

[36] Kwon J, Park JH, Ku S, Jeong Y, Paik NJ, Park YL (2019). A soft wearable robotic ankle-foot-orthosis for post-stroke patients. IEEE Robot Autom Lett.

[37] Liu Z, Zhang P, Yan M, Xie YM, Huang GZ (2019). Additive manufacturing of specific ankle-foot orthoses for persons after stroke: a preliminary study based on gait analysis data. Math Biosci Eng.

[38] (2021). Global strategy on digital health 2020-2025. World Health Organization.

[39] Ashraf M, Choudhary N, Kamboh UA (2022). Early experience with patient-specific low-cost 3D-printed polymethylmethacrylate cranioplasty implants in a lower-middle-income-country: technical note and economic analysis. Surg Neurol Int.

[40] Woodson T, Alcantara JT, do Nascimento MS (2019). Is 3D printing an inclusive innovation?: an examination of 3D printing in Brazil. Technovation.

[41] Nakipoğlu Yüzer GF, Koyuncu E, Çam P, Özgirgin N (2018). The regularity of orthosis use and the reasons for disuse in stroke patients. Int J Rehabil Res.

[42] Liz L, da Silva TG, Michaelsen SM (2023). Validity, reliability, and measurement error of the remote Fugl-Meyer Assessment by videoconferencing: Tele-FMA. Phys Ther.

[43] Quinn TJ, Langhorne P, Stott DJ (2011). Barthel index for stroke trials: development, properties, and application. Stroke.

[44] Kumar A, Schmeler MR, Karmarkar AM (2013). Test-retest reliability of the functional mobility assessment (FMA): a pilot study. Disabil Rehabil Assist Technol.

[45] Khramtsov DM, Stoyanov ОМ, Hruzevskyi ОА, Shaevchuk HY (2022). Complex neurorehabilitation of post-stroke patients. Med Sci Ukraine.

[46] Akter S (2018). Measurement of hand grip strength by digital Jamar hand dynamometer: a study on CRP staffs & BHPI students [Dissertation]. http://library.crp-bangladesh.org:8080/xmlui/handle/123456789/520.

[47] Bertrand AM, Fournier K, Wick Brasey MG, Kaiser ML, Frischknecht R, Diserens K (2015). Reliability of maximal grip strength measurements and grip strength recovery following a stroke. J Hand Ther.

[48] Sánchez-Aranda L, Fernández-Ortega J, Martín-Fuentes I (2024). Reliability and criterion validity of a low-cost handgrip dynamometer: the Camry. medRxiv.

[49] Hobart JC, Thompson AJ (2001). The five item Barthel index. J Neurol Neurosurg Psychiatry.

[50] Taghizadeh G, Martinez-Martin P, Meimandi M (2020). Barthel Index and modified Rankin Scale: psychometric properties during medication phases in idiopathic Parkinson disease. Ann Phys Rehabil Med.

[51] Sakamoto D, Hamaguchi T, Nakayama Y, Abo M (2026). Reliability and validity of a newly developed Action Research Arm Test for upper limb function assessment in patients with stroke: a comparison with the conventional version. PLoS One.

[52] Tasseel-Ponche S, Delafontaine A, Godefroy O (2022). Walking speed at the acute and subacute stroke stage: a descriptive meta-analysis. Front Neurol.

[53] Peters DM, Fritz SL, Krotish DE (2013). Assessing the reliability and validity of a shorter walk test compared with the 10-Meter Walk Test for measurements of gait speed in healthy, older adults. J Geriatr Phys Ther.

[54] Desai N (2021). Addressing the gaps in assessment and treatment of the upper extremity in rehabilitation of individuals with neurological conditions [PhD thesis]. https://utoronto.scholaris.ca/server/api/core/bitstreams/7da1c076-ef0a-4b08-88f0-03c44634e232/content.

