# Supplementary material for: The Use of 3D Printing Technology in Rehabilitation for Adults Living With Neurological Conditions: Scoping Review
Source: JMIR Rehabil Assist Technol. 2026 May 6;13:e81782. doi: 10.2196/81782 (PMC13148325; doi:10.2196/81782)
Supplement: Multimedia Appendix 1 [file rehab-v13-e81782-s001.docx]

| **Low-Income Countries (GNI per capita: < $1,145 in 2023)** | **Lower-Middle-Income Countries (GNI per capita: $1,146 - $4,515 in 2023)** | **Upper-Middle Income Countries (GNI per capita: $4,516 and $14,005 in 2023)** | **Higher-Income Countries (GNI per capita: > $14,005 in 2023)** |
| --- | --- | --- | --- |
| Burkina Faso | Bangladesh | Albania | Australia |
| Ethiopia | Benin | Argentina | Austria |
| Guinea | Bolivia | Armenia | Bahrain |
| Madagascar | Cambodia | Azerbaijan | Belgium |
| Malawi | Cameron | Belarus | Brunei |
| Mozambique | Cote d’Ivoire | Botswana | Canada |
| Rwanda | Egypt | Brazil | Chile |
| Togo | El Salvador | Bulgaria | Croatia |
| Uganda | Ghana | China | Cyprus |
| Zambia | Honduras | Colombia | Czechia |
|  | India | Costa Rica | Denmark |
|  | Indonesia | Dominican Republic | Estonia |
|  | Iran | Ecuador | Finland |
|  | Kenya | Georgia | France |
|  | Kyrgyzstan | Guatemala | Germany |
|  | Laos | Jamacia | Greece |
|  | Mongolia | Jordon | Hong Kong |
|  | Morocco | Kazakhstan | Hungary |
|  | Nepal | Malaysia | Ireland |
|  | Nicaragua | Mauritius | Israel |
|  | Nigeria | Mexico | Italy |
|  | Pakistan | Namibia | Japan |
|  | Philippines | Paraguay | Kuwait |
|  | Senegal | Peru | Lativa |
|  | Sri Lanka | Russia | Luxembourg |
|  | Tajikistan | South Africa | Malta |
|  | Tanzania | Thailand | Netherlands |
|  | Tunisia | Turkey | New Zealand |
|  | Ukraine | Venezuela | Norway |
|  | Vietnam |  | Oman |
|  | Zimbabwe |  | Panama |
|  |  |  | Poland |
|  |  |  | Portugal |
|  |  |  | Qatar |
|  |  |  | Romania |
|  |  |  | Saudi Arabia |
|  |  |  | Singapore |
|  |  |  | Slovakia |
|  |  |  | Slovenia |
|  |  |  | South Korea |
|  |  |  | Spain |
|  |  |  | Sweden |
|  |  |  | Switzerland |
|  |  |  | Taiwan |
|  |  |  | Trinidad and Tobago |
|  |  |  | United Arab Emirates |
|  |  |  | United Kingdom |
|  |  |  | United States |
|  |  |  | Uruguay |
