# Supplementary material for: The Use of 3D Printing Technology in Rehabilitation for Adults Living With Neurological Conditions: Scoping Review
Source: JMIR Rehabil Assist Technol. 2026 May 6;13:e81782. doi: 10.2196/81782 (PMC13148325; doi:10.2196/81782)
Supplement: Multimedia Appendix 2 [file rehab-v13-e81782-s002.pdf]

<table>
<tr><th>Databases</th><th>Search Strategy</th><th>Filters</th></tr>
<tr><td>MEDLINE (PubMed), Nursing and Allied Health Premium, Web of Science</td><td>(3D printing) AND (rehabilitation OR physiotherapy OR physical therapy OR physical rehabilitation)</td><td>English, Last 5 years (2019-2024)</td></tr>
<tr><th colspan="3">Expanded Boolean Logic (MEDLINE)</th></tr>
<tr><td colspan="3">(("printing, three dimensional"[MeSH Terms] OR ("printing"[All Fields] AND "three dimensional"[All Fields]) OR "three-dimensional printing"[All Fields] OR ("3d"[All Fields] AND "printing"[All Fields]) OR "3d printing"[All Fields]) AND ("rehabilitant"[All Fields] OR "rehabilitants"[All Fields] OR "rehabilitate"[All Fields] OR "rehabilitated"[All Fields] OR "rehabilitates"[All Fields] OR "rehabilitating"[All Fields] OR "rehabilitation"[MeSH Terms] OR "rehabilitation"[All Fields] OR "rehabilitations"[All Fields] OR "rehabilitative"[All Fields] OR "rehabilitation"[MeSH Subheading] OR "rehabilitation s"[All Fields] OR "rehabilitational"[All Fields] OR "rehabilitator"[All Fields] OR "rehabilitators"[All Fields] OR ("physical therapy modalities"[MeSH Terms] OR ("physical"[All Fields] AND "therapy"[All Fields] AND "modalities"[All Fields]) OR "physical therapy modalities"[All Fields] OR "physiotherapies"[All Fields] OR "physiotherapy"[All Fields]) OR ("physical therapy modalities"[MeSH Terms] OR ("physical"[All Fields] AND "therapy"[All Fields] AND "modalities"[All Fields]) OR "physical therapy modalities"[All Fields] OR ("physical"[All Fields] AND "therapy"[All Fields]) OR "physical therapy"[All Fields]) OR (("physical examination"[MeSH Terms] OR ("physical"[All Fields] AND "examination"[All Fields]) OR "physical examination"[All Fields] OR "physical"[All Fields] OR "physically"[All Fields] OR "physicals"[All Fields]) AND ("rehabilitant"[All Fields] OR "rehabilitants"[All Fields] OR "rehabilitate"[All Fields] OR "rehabilitated"[All Fields] OR "rehabilitates"[All Fields] OR "rehabilitating"[All Fields] OR "rehabilitation"[MeSH Terms] OR "rehabilitation"[All Fields] OR "rehabilitations"[All Fields] OR "rehabilitative"[All Fields] OR "rehabilitation"[MeSH Subheading] OR "rehabilitation s"[All Fields] OR "rehabilitational"[All Fields] OR "rehabilitator"[All Fields] OR "rehabilitators"[All Fields])))) AND ((fft[Filter]) AND (english[Filter]))</td></tr>
<tr><th colspan="3">Translations (MEDLINE)</th></tr>
<tr><td>3D printing</td><td colspan="2">"printing, three-dimensional"[MeSH Terms] OR ("printing"[All Fields] AND "three-dimensional"[All Fields]) OR "three-dimensional printing"[All Fields] OR ("3d"[All Fields] AND "printing"[All Fields]) OR "3d printing"[All Fields]</td></tr>
<tr><td>Physiotherapy</td><td colspan="2">"physical therapy modalities"[MeSH Terms] OR ("physical"[All Fields] AND "therapy"[All Fields] AND "modalities"[All Fields]) OR "physical therapy modalities"[All Fields] OR "physiotherapies"[All Fields] OR "physiotherapy"[All Fields]</td></tr>
<tr><td>Physical Therapy</td><td colspan="2">"physical therapy modalities"[MeSH Terms] OR ("physical"[All Fields] AND "therapy"[All Fields] AND "modalities"[All Fields]) OR "physical therapy modalities"[All Fields] OR ("physical"[All Fields] AND "therapy"[All Fields]) OR "physical therapy"[All Fields]</td></tr>
</table>

Physical	"physical examination"[MeSH Terms] OR ("physical"[All Fields] AND "examination"[All Fields]) OR "physical examination"[All Fields] OR "physical"[All Fields] OR "physically"[All Fields] OR "physicals"[All Fields]
Rehabilitation	"rehabilitant"[All Fields] OR "rehabilitant's"[All Fields] OR "rehabilitants"[All Fields] OR "rehabilitate"[All Fields] OR "rehabilitated"[All Fields] OR "rehabilitates"[All Fields] OR "rehabilitating"[All Fields] OR "rehabilitation"[MeSH Terms] OR "rehabilitation"[All Fields] OR "rehabilitations"[All Fields] OR "rehabilitative"[All Fields] OR "rehabilitation"[Subheading] OR "rehabilitation's"[All Fields] OR "rehabilitational"[All Fields] OR "rehabilitator"[All Fields] OR "rehabilitators"[All Fields]
