# Supplementary material for: The Use of 3D Printing Technology in Rehabilitation for Adults Living With Neurological Conditions: Scoping Review
Source: JMIR Rehabil Assist Technol. 2026 May 6;13:e81782. doi: 10.2196/81782 (PMC13148325; doi:10.2196/81782)
Supplement: Multimedia Appendix 3 [file rehab-v13-e81782-s003.pdf]

Label	Instructions to extractors
General characteristics	
Author(s)	Eg, John; John & Doe; John et al. (for more than 2 authors)
Title	Title of the article
Publication (year)	The year the article was published.
Date data were collected	Ie, Month, Year
Study type/source	Eg, RCT, systematic review, etc
Demographics	
Country	Low-Income Countries (GNI per capita: < $1,145 in 2023) Burkina Faso Ethiopia Guinea Madagascar Malawi Mozambique Rwanda Togo Uganda Zambia<br><br>Lower-Middle-Income Countries (GNI per capita: $1,146 - $4,515 in 2023) Bangladesh Benin Bolivia Cambodia Cameron Cote d'Ivoire Egypt El Salvador Ghana Honduras India Indonesia Iran Kenya Kyrgyzstan Laos Mongolia Morocco Nepal Nicaragua Nigeria Pakistan Philippines Senegal Sri Lanka Tajikistan Tanzania Tunisia Ukraine Vietnam Zimbabwe<br><br>Upper-Middle Income Countries (GNI per capita: $4,516 and $14,005 in 2023) Albania Argentina Armenia Azerbaijan Belarus Botswana Brazil Bulgaria China Colombia Costa Rica Dominican Republic Ecuador Georgia Guatemala Jamacia Jordon Kazakhstan Malaysia Mauritius Mexico Namibia Paraguay Peru Russia South Africa Thailand Turkey Venezuela<br><br>Higher-Income Countries (GNI per capita: > $14,005 in 2023) Australia Austria Bahrain Belgium Brunei Canada Chile Croatia Cyprus Czechia Denmark Estonia Finland France Germany Greece Hong Kong Hungary Ireland Israel Italy Japan Kuwait Lativa Luxembourg Malta Netherlands New Zealand Norway Oman Panama Poland Portugal Qatar Romania Saudi Arabia Singapore Slovakia Slovenia South Korea Spain Sweden Switzerland Taiwan Trinidad and Tobago

	United Arab Emirates United Kingdom<br>United States Uruguay
Aim/purpose	Primary objective of the study (eg, "To evaluate the effectiveness of 3D-printed wrist orthoses in improving motor function for stroke survivors")
Population	Who? Exclude based on inclusion/exclusion criteria. (refer to PCC Framework).
Sample size	Total - and if applicable include control group and experimental group sizes
Age (yrs)	Age of participants
Gender	Gender of participants
Intervention	
Setting	Clinical, home-based, or laboratory rehabilitation settings
Neurological condition	Stroke, Parkison Disease, Spinal Nerve Injury, etc.
Type of intervention (Technology)	Orthotics, insoles, exoskeleton, robotics, etc.
Type of intervention (3D Printed Details/Materials and Processes)	PLA, TPU, ABS, PA-12 (nylon), composite materials, variable-stiffness materials, etc.; FDM, SLS, PolyJet, Multi Jet Fusion, etc.
Type of intervention (Anatomical Focus)	Upper Extremities or Lower Extremities
Duration of the intervention	Time
Outcomes	
Outcome summary	Summary of functional outcomes (eg, improvements in grip strength, dexterity, range of motion, spasticity, gait, etc.)
Outcome measures	Types of assessments used to evaluate motor recovery outcomes (Ie, standardized clinical scales, functional performance tests, biomechanical measurements)
