# Supplementary figures and images for: The Use of 3D Printing Technology in Rehabilitation for Adults Living With Neurological Conditions: Scoping Review
Source: JMIR Rehabil Assist Technol. 2026 May 6;13:e81782. doi: 10.2196/81782 (PMC13148325; doi:10.2196/81782)

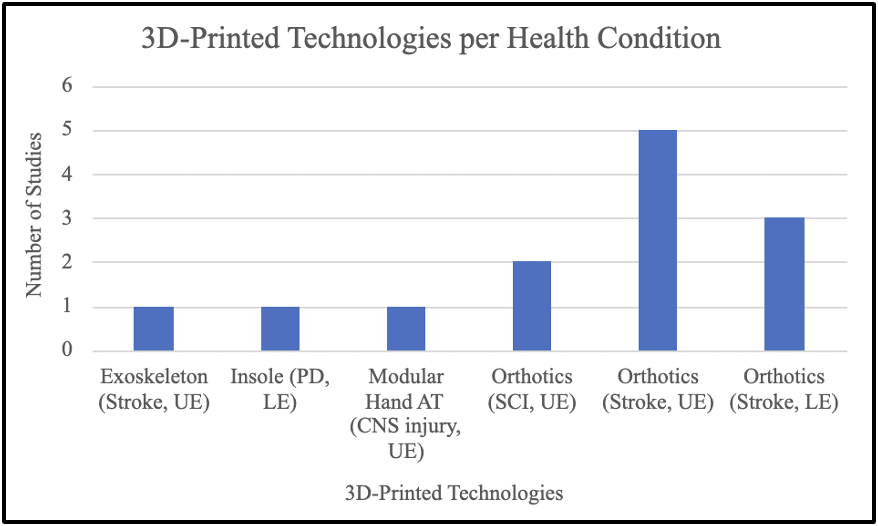

Supplement: Multimedia Appendix 4 [file rehab-v13-e81782-s004.png]
