# Supplementary material for: The Use of 3D Printing Technology in Rehabilitation for Adults Living With Neurological Conditions: Scoping Review
Source: JMIR Rehabil Assist Technol. 2026 May 6;13:e81782. doi: 10.2196/81782 (PMC13148325; doi:10.2196/81782)
Supplement: Checklist 1 [file rehab-v13-e81782-s005.pdf]

Preferred Reporting Items for Systematic reviews and Meta-Analyses extension for Scoping Reviews (PRISMA-ScR) Checklist

SECTION	ITEM	PRISMA-ScR CHECKLIST ITEM	REPORTED ON PAGE #
**TITLE**			
Title	1	The Use of 3D Printing Technology in Rehabilitation for Adults Living With Neurological Conditions: Scoping Review	1
**ABSTRACT**			
Structured summary	2	**Background:** Neurorehabilitation plays a key role in improving motor recovery for people with neurological conditions. Although 3D printing has emerged as a promising rehabilitation tool, little is known on how it is used for the rehabilitation of adults living with neurological conditions worldwide.<br>**Objective:** We aimed to provide a comprehensive overview of 3D printing in neurorehabilitation and precisely explore how it is used to improve motor recovery for adults with neurological conditions living in higher- and lower-middle-income countries.<br>**Methods:** We conducted a scoping review following the Joanna Briggs Institute guidelines. After searching 3 databases (MEDLINE, Web of Science, and Nursing and Allied Health Premium), 2 independent reviewers screened and selected English-language studies involving adults (≥18 years) published between 2019 and 2024 to capture the most recent advancements in this field. We extracted relevant information on neurological conditions, motor recovery outcomes, and types of 3D printing and offered a comparative analysis of 3D printing in physical neurorehabilitation from the perspective of national income levels using a modified Joanna Briggs Institute extraction form. We synthesized the findings narratively with tabular support.<br>**Results:** After screening 2752 titles and abstracts and 103 (3.7%) full texts, we included 13 (0.5%) studies based on our inclusion criteria. All included studies were conducted in upper-middle–income or high-income countries, and most studies (9/13, 69.2%) focused on stroke, followed by spinal cord injury (2/13, 15.4%), Parkinson disease (1/13, 7.7%), and central nerve disease (1/13, 7.7%). The 3D-printed rehabilitation tools included orthotics (7/13, 53.8% for the upper extremities [UEs]; 3/13, 23.1% for the lower extremities [LEs]), an exoskeleton (1/13, 7.7%; UEs), a modular assistive hand device (1/13, 7.7%; UEs), and an insole (1/13, 7.7%; LEs). In total, 69.2% (9/13) of the studies targeted UE rehabilitation, measured using the Action Research Arm Test, active range of motion, the box and block test, the Fugl-Meyer Assessment, the Modified Ashworth Scale, the manual function test, range of motion, and the Toronto Rehabilitation Institute Hand Function Test, and 30.8% (4/13) targeted LE rehabilitation, measured using the 10-m walk test, anteroposterior ground reaction force	1

St. Michael's
Inspired Care.
Inspiring Science.

SECTION	ITEM	PRISMA-ScR CHECKLIST ITEM	REPORTED ON PAGE #
		analysis, the Barthel index, the Tinetti scale, the RehaWatch system, and the GaitWatch system. **Conclusions:** Used as a rehabilitation tool, 3D printing technology has demonstrated significant potential in improving upper and lower motor recovery for people with certain neurological conditions in high-middle–income countries. Future research should explore the implementation feasibility and effectiveness of these technologies across different neurological conditions and income settings, particularly in low- and lower-middle–income countries.	
**INTRODUCTION**			
Rationale	3	According to a 2021 report published in *The Lancet Neurology* by the World Health Organization (WHO), over 3 billion people in the world are living with a neurological condition [2,3].<br><br>In the United States alone, the financial and public health burden of neurological conditions is estimated to cost US $1.7 trillion annually [1]. Given the global prevalence of neurological conditions, and with over 80% of neurological-related deaths and health losses occurring in low- and middle-income countries (LMICs), there is a need for effective treatment strategies to improve patient health outcomes and reduce associated burdens worldwide [1-7].<br><br>Worldwide, 3D printing has the potential to improve access to rehabilitation devices in lower-middle–income countries where traditional manufacturing processes can be both costly and time-consuming [14,15].	2
Objectives	4	The objective of our scoping review was to provide a comprehensive overview of how 3D printing technologies have recently been used in neurorehabilitation for adults. We aimed to document and compare their applications across high-income, upper-middle–income, lower-middle–income, and low-income countries and identify the types of assessments used to evaluate motor recovery outcomes when using 3D-printed rehabilitation tools, including standardized clinical scales, functional performance tests, and biomechanical measurements. To our knowledge, this study is the first to offer a comparative analysis of 3D printing in physical neurorehabilitation from the perspective of national income levels.	2
**METHODS**			
Protocol and registration	5	We conducted this scoping review in accordance with the Joanna Briggs Institute (JBI) methodology for scoping reviews [17] and aimed to answer the following question: how is 3D	2

SECTION	ITEM	PRISMA-ScR CHECKLIST ITEM	REPORTED ON PAGE #
		printing used to improve motor recovery for adults with neurological conditions in higher- and lower-middle–income countries?	
Eligibility criteria	6	We used the population, concept, and context framework as recommended by the JBI guidelines [17]. We included studies involving adult participants (18 years and older) with neurological conditions who received rehabilitation interventions aimed at improving or restoring motor function. Participants under 18 years of age were excluded as pediatric populations have different developmental trajectories and rehabilitation needs, which fall outside the scope of this review [18]. Eligible conditions included acquired brain injuries, neurodegenerative disorders, and traumatic brain or spinal cord injuries [19].<br><br>The concept for this review focused on the application of 3D printing technology in improving motor recovery for adults with neurological conditions.<br><br>Rehabilitation interventions included physiotherapy or physical rehabilitation strategies designed to enhance motor abilities.<br><br>Settings included clinical environments (eg, hospitals, rehabilitation centers, and outpatient clinics), home-based rehabilitation programs, and laboratory-based rehabilitation programs.<br><br>This scoping review considered studies conducted in contexts of higher-income, upper-middle–income, lower-middle–income, and low-income countries as defined by the World Bank based on gross national income per capita for 2023 (Multimedia Appendix 1) [20].<br><br>No limitations were placed on the gender, ethnicity, or religion of the participants included in the studies.	2-3
Information sources*	7	We developed a rigorous search strategy in 3 steps. First, we conducted a preliminary search of MEDLINE (PubMed) to identify articles on the topic and define main keywords. In this preliminary search, we used the keywords “3D printing” and “rehabilitation” to capture as many articles on the topic as possible, thus providing insights into existing literature. We then reviewed keywords contained in titles and abstracts and the indexing terms used to describe potentially included articles (reviewing keywords and indexing terms from relevant titles and abstracts), which was then used to refine our search strategy. Second, we developed a refined search strategy incorporating additional keywords: “physiotherapy,” “physical therapy,” or	3

SECTION	ITEM	PRISMA-ScR CHECKLIST ITEM	REPORTED ON PAGE #
		"physical rehabilitation" (Multimedia Appendix 2). This refined search strategy incorporated both MeSH (Medical Subject Headings) terms and text word searches in MEDLINE (PubMed) and was adapted using equivalent text word strategies in 2 other databases (Web of Science and Nursing and Allied Health Premium). In this step, a filter for English-language publications was applied across all databases to maintain consistency (Multimedia Appendix 2). Third, the reference lists of all articles included in the review were manually screened for additional relevant papers. This step aimed to capture any pertinent studies that may not have been identified through the database searches.	
Search	8	(nested table below)	Multimedia Appendix 2

Databases	Search Strategy	Filters
MEDLINE (PubMed), Nursing and Allied Health Premium, Web of Science	(3D printing) AND (rehabilitation OR physiotherapy OR physical therapy OR physical rehabilitation)	English, Last 5 years (2019-2024)
**Expanded Boolean Logic (MEDLINE)**		
(("printing, three dimensional"[MeSH Terms] OR ("printing"[All Fields] AND "three dimensional"[All Fields]) OR "three-dimensional printing"[All Fields] OR ("3d"[All Fields] AND "printing"[All Fields]) OR "3d printing"[All Fields]) AND ("rehabilitant"[All Fields] OR "rehabilitants"[All Fields] OR "rehabilitate"[All Fields] OR "rehabilitated"[All Fields] OR "rehabilitates"[All Fields] OR "rehabilitating"[All Fields] OR "rehabilitation"[MeSH Terms] OR "rehabilitation"[All Fields] OR "rehabilitations"[All Fields] OR "rehabilitative"[All Fields] OR "rehabilitation"[MeSH Subheading] OR "rehabilitation s"[All Fields] OR "rehabilitational"[All Fields] OR "rehabilitator"[All Fields] OR "rehabilitators"[All Fields] OR ("physical therapy modalities"[MeSH Terms] OR ("physical"[All Fields] AND "therapy"[All Fields] AND "modalities"[All Fields]) OR "physical therapy modalities"[All Fields] OR "physiotherapies"[All Fields] OR "physiotherapy"[All Fields]) OR ("physical therapy modalities"[MeSH Terms] OR ("physical"[All Fields] AND "therapy"[All Fields] AND "modalities"[All Fields]) OR "physical therapy modalities"[All Fields] OR ("physical"[All Fields] AND "therapy"[All Fields]) OR "physical therapy"[All Fields]) OR (("physical examination"[MeSH Terms] OR ("physical"[All Fields] AND "examination"[All Fields]) OR "physical examination"[All Fields] OR "physical"[All Fields] OR "physically"[All Fields] OR "physicals"[All Fields]) AND ("rehabilitant"[All Fields] OR "rehabilitants"[All Fields] OR "rehabilitate"[All Fields]		

SECTION	ITEM	PRISMA-ScR CHECKLIST ITEM	REPORTED ON PAGE #
		OR "rehabilitated"[All Fields] OR "rehabilitates"[All Fields] OR "rehabilitating"[All Fields] OR "rehabilitation"[MeSH Terms] OR "rehabilitation"[All Fields] OR "rehabilitations"[All Fields] OR "rehabilitative"[All Fields] OR "rehabilitation"[MeSH Subheading] OR "rehabilitation s"[All Fields] OR "rehabilitational"[All Fields] OR "rehabilitator"[All Fields] OR "rehabilitators"[All Fields])))) AND ((fft[Filter]) AND (english[Filter]))	
Selection of sources of evidence†	9	Following the 3-database search, we collated all identified studies and used Mendeley Reference Manager (version 2.51.0; Elsevier) and Covidence (Veritas Health Innovation) for citation management and removal of duplicates. Titles and abstracts were screened by 2 independent reviewers (SA and ZL) for assessment against the inclusion and exclusion criteria. We screened studies published from 2019 onward, with the final search completed on November 21, 2024, to capture the most recent advancements in 3D printing technologies for neurorehabilitation. We included a variety of study designs (ie, randomized controlled trials [RCTs], nonrandomized experimental studies, pilot and feasibility studies, case studies, and systematic reviews) in our search to capture both the effectiveness of the interventions and the practical considerations surrounding their implementation. RCTs and non-RCTs were included to assess potential causal relationships between 3D printing interventions and motor recovery outcomes [21]. Pilot and feasibility studies were eligible if they examined the usability, acceptability, or logistical feasibility of 3D-printed devices in rehabilitation contexts [22]. Case studies were included if they provided detailed examples of individualized application [23]. Systematic reviews were considered if they synthesized primary research aligned with our inclusion criteria [24]. Opinion pieces, editorials, commentaries, and protocols without full-text availability were excluded as they did not contribute to the empirical evidence required for conclusions. When the inclusion criteria were met at the title and abstract screening stage, the full texts were screened through the same 2-reviewer screening method. Any disagreements were resolved through consensus with a third reviewer (JON) when necessary.	3
Data charting process‡	10	We extracted relevant data from the included full-text studies. We used a data extraction form that was developed based on the JBI guidelines (Multimedia Appendix 3) to ensure completeness of data extraction.	3, Multimedia Appendix 3
Data items	11	The extraction form included (1) general study characteristics, including authors, title, year of publication, date of data collection, study type, country, and study aim; (2) population details, such as sample size, age, gender, and the health condition being addressed; (3) intervention details, including setting (eg, clinical, home based, or laboratory) and duration of the intervention, type of 3D-printed technology used (eg, orthosis or insole), stiffness and type of	3, Multimedia Appendix 3

SECTION	ITEM	PRISMA-ScR CHECKLIST ITEM	REPORTED ON PAGE #
		polymer, neurological condition (eg, stroke or Parkinson disease), and motor area focus (upper extremities [UEs] or lower extremities [LEs]); and (4) outcomes, including the types of assessments used to evaluate motor recovery, such as standardized clinical scales, functional performance tests, and biomechanical measurements.	
Critical appraisal of individual sources of evidence§	12	N/A	N/A
Synthesis of results	13	We analyzed the data using descriptive qualitative content analysis and synthesized the extracted data from a geographical perspective. Data were systematically mapped based on the objectives of our scoping review to capture study characteristics, (authors, title, and publication year), the type of 3D-printing technology used, the associated neurological condition and rehabilitation goal, outcome measures, and country of implementation. A comparative approach was applied to assess differences between high-income and lower-middle–income countries, documenting disparities in access (as reported through the availability of 3D printing technology, affordability considerations, descriptions of infrastructure, and integration into clinical practice) and effectiveness. Findings were synthesized into thematic categories, providing a comprehensive overview of how 3D-printed technologies have been used to support motor recovery in different health care contexts.	3-4
**RESULTS**			

SECTION	ITEM	PRISMA-ScR CHECKLIST ITEM	REPORTED ON PAGE #
Selection of sources of evidence	14	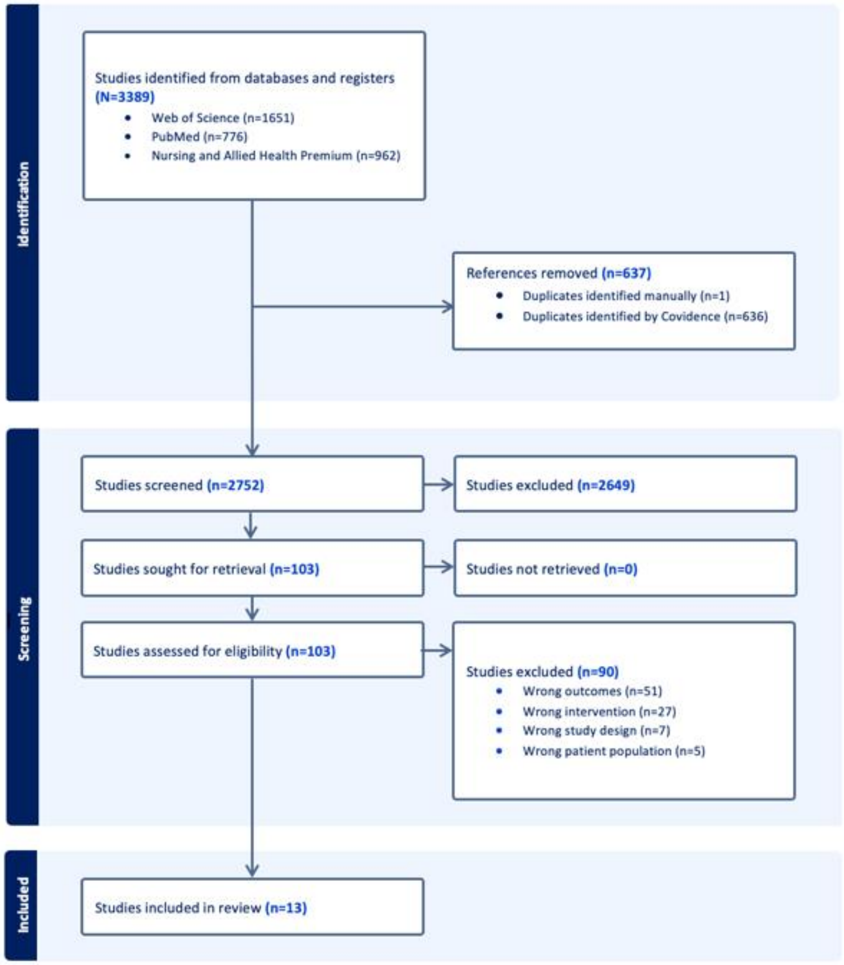	5
Characteristics of sources of evidence	15	A total of 2752 titles and abstracts were screened. Of these 2752 articles, after removing 637 (23.1%) duplicates, 103 (3.7%) full-text articles were assessed for eligibility, and 90 (3.3%) were excluded (Figure 1). We included 13 studies [25-37], with sample sizes ranging from single case reports (N=1 individual) to small trials (N=31 individuals) (Table 1). The reported	4,7, Multimedia Appendix 4

SECTION	ITEM	PRISMA-ScR CHECKLIST ITEM	REPORTED ON PAGE #
		participant ages ranged from 23 to 83 years, aligning with the inclusion criterion of adult participants (18 years and older).<br>All included studies [25-37] were conducted in upper-middle–income or high-income countries (ie, China, South Korea, the United States, Spain, and Hungary; Figure 2). While 4 different types of neurorehabilitation tools were designed using 3D printing—orthotics (10/13, 76.9%) [25,26,28,30-33,35-37], an exoskeleton (1/13, 7.7%) [27], a modular assistive hand technology device (1/13, 7.7%) [29], and an insole (1/13, 7.7%) [34] (Multimedia Appendix 4)—most included studies (8/13, 61.5%) primarily focused on orthoses for stroke rehabilitation [25,26,28,30-32,35-37].	
Critical appraisal within sources of evidence	16	N/A	N/A
Results of individual sources of evidence	17	**Upper-extremity impairments**<br><br>Chen et al [25], 2022, China<br>Stroke<br>Hand splint<br>PLA[a]; 3D printing method and process not specified<br>Improved grip and lateral pinch strength; no significant change in palmar pinch and gross movement<br>ARAT[b] and pinch force tests[c]<br><br>Demeco et al [26], 2023, country not specified<br>Stroke<br>Hand splint<br>Not specified<br>Improved upper-limb ROM[d], hand dexterity, and pinch strength (palmar and lateral); reduced spasticity<br>ROM, BBT[e], pinch force tests, FMA[f], and MAS[g]<br><br>Dudley et al [27], 2021, United States<br>Stroke<br>Exoskeleton	6-7

SECTION	ITEM	PRISMA-ScR CHECKLIST ITEM	REPORTED ON PAGE #
		PLA+1% copper additive (PLACTIVE); FDM[h] (Ultimaker 2+ Extended); infill: 35%-40%; layer height: 0.15-0.25 mm; bed: 50 °C; shell: 0.8 mm; print speed: 60-100 mm/s<br>Improved finger flexion and extension, grasp function, and hand dexterity<br>FMA and BBT<br><br>Huber et al [28], 2023, United States<br>Stroke<br>Hand splint<br>Variable-stiffness TPU[i] powder; SLS[j] 3D printing; printer and parameters not reported<br>Improved hand dexterity and pincer force; no significant change in pincer aperture<br>Pincer force test, pincer aperture test, and BBT<br><br>Kuo et al [29], 2024, China<br>Neurological central nerve injury<br>Modular assistive hand technology device<br>TPU filament (1.75 mm); FDM (Original Prusa i3 MK3S+); CAD[k] designed (SolidWorks, DesignSpark Mechanical, and Meshmixer)<br>Improved shoulder AROM[l] and functional hand tasks; no significant change in hand dexterity or grip strength<br>AROM, BBT, Jamar dynamometer, and functional hand tasks<br><br>Yeh et al [30], 2023, China<br>Spinal cord injury<br>Hand splint<br>Material not specified; FDM (FlashForge Finder); layer height: 0.2 mm; infill: 15%; speed: 60-80 mm/s; print temperature: 200 °C; nonprinted components: silicone finger cots; acrylic linkages (3 mm) laser cut<br>Improved pinch force and hand dexterity<br>Pinch force test and BBT<br><br>Toth et al [31], 2020, Hungary<br>Stroke<br>Hand splint<br>PA[m] 2200 (nylon) framework; TPU sheets; nitinol SMA[n]+ Kapton heating elements; SLS (EOS Formiga P 110); 110 µm layers; FDM; integrated heating elements	

SECTION	ITEM	PRISMA-ScR CHECKLIST ITEM	REPORTED ON PAGE #
		Improved pencil holding, handle and tool grasp, vertical handle grasp, cup holding, door handle operation, and device assembly; no significant improvement in eraser holding<br>MFT[o]<br><br>Yang et al [32], 2021, China<br>Stroke<br>Hand splint<br>ABS[p] filament; FDM (UP Box); layer thickness: 0.2 mm; infill: 20%; nozzle temperature: 230 °C; print speed: 60 mm/s<br>Improved upper-extremity motor function; reduced spasticity<br>MAS and FMA<br><br>Yoo et al [33], 2019, South Korea<br>Spinal cord injury<br>Hand splint<br>PLA; FDM (Moment 2); CAD designed (SolidWorks); postprint heat reshaping; nonprinted components: self-adhesive padding, Velcro, and nylon thread<br>Improved grasp, lift, and object manipulation<br>TRI-HFT[q]<br><br>**Lower-extremity impairments**<br><br>Brognara et al [34], 2020, Spain<br>Parkinson disease<br>Insole<br>Material not specified; 3D foot scan (3D Sense); CAD designed (Rhinoceros 3D); FDM (Delta WASP 4070); custom insole with 2 blunted cones (plantar stimulation)<br>Improved balance; no significant change in daily activities<br>Barthel index and Tinetti scale<br><br>Hsu et al [35], 2020, China<br>Stroke<br>Ankle-foot orthoses<br>Nylon (4611); 3D scan (Structure Sensor; OBJ[t]→STL[s]); FDM (Ultimaker); approximately 3-mm wall thickness	

SECTION	ITEM	PRISMA-ScR CHECKLIST ITEM	REPORTED ON PAGE #
		Improved gait parameters; no significant change in walking speed<br>RehaWatch system and 10MWT[u]<br><br>Kwon et al [36], 2019, China<br>Stroke<br>Ankle-foot orthoses<br>TPU filament; VeroBlackPlus (rigid components); FDM (Cubicon Single Plus); PolyJet (Objet 30 Prime)<br>Improved paretic propulsion; no significant change in walking speed<br>Anteroposterior ground reaction force analysis and 10MWT<br><br>Liu et al [37], 2019, South Korea<br>Stroke<br>Ankle-foot orthoses<br>PA-12; 3D scan (EinScan-Pro); model modification (Geomagic Studio); MJF[v]<br>Increased gait velocity and stride length; no significant changes in cadence and step symmetry<br>GaitWatch system	
Synthesis of results	18	Across studies, outcome measures were predominantly used in pretest-posttest designs to assess changes in motor performance before and after the application of 3D-printed rehabilitation tools. The most frequently used standardized outcome measures for UEs were the pinch force test (4/13, 30.8%) [25,26,28,30] and the box and block test (5/13, 38.5%) [26-30]. For LEs, a commonly used outcome measure was the 10-m walk test (10MWT; 2/13, 15.4%) [35,36].	8
**DISCUSSION**			
Summary of evidence	19	To our knowledge, this scoping review is the first to explore the application of 3D printing rehabilitation interventions for adults with neurological conditions from a geographical perspective. We selected a geographical perspective and the World Bank national income classification to reflect the socioeconomic and structural determinants of adoption that may influence whether 3D-printed neurorehabilitation devices can be implemented across international health systems. Our geographical analysis showed that rehabilitation research on	8-9

SECTION	ITEM	PRISMA-ScR CHECKLIST ITEM	REPORTED ON PAGE #
		3D printing technologies is primarily concentrated in higher and upper-middle–income countries (ie, China, the United States, South Korea, Spain, and Hungary).<br><br>Our scoping review synthesized evidence on the application of novel technology, including 3D-printed orthotics, exoskeletons, modular assistive hand devices, and insoles, supporting motor rehabilitation for individuals who have experienced a stroke or spinal cord injury or been diagnosed with Parkinson disease or central nerve injury. Among the technologies reviewed, orthotic devices emerged as the most widely studied, primarily addressing lower motor impairments for persons who experienced a stroke.<br><br>Results from our scoping review identified 4 gold-standard outcome measures being used for assessing the impact of 3D printing technologies on UE rehabilitation: the Fugl-Meyer Assessment (FMA), the Action Research Arm Test (ARAT), the Jamar dynamometer, and the Barthel index. For LE rehabilitation, the 10MWT was the only gold-standard outcome measure identified.	
Limitations	20	Although previous research has demonstrated improvements in motor recovery outcomes through 3D-printed rehabilitation technologies, several limitations remain. Studies included in this review had small sample sizes and heterogeneous intervention protocols, limiting the ability to draw generalizable conclusions. Additionally, the search strategy may have inadvertently excluded relevant studies by not expanding the keywords to include other discipline-specific terminology (eg, “occupational therapy,” “audiology,” and “nursing”). Not indexing these terms potentially narrowed the scope of the findings. Moreover, restricting inclusion to English-language publications may have introduced a language bias and limited the generalizability of the results. All eligible studies were conducted in high- or middle-income countries, which limited comparisons across income groups and may have contributed to heterogeneity as the effectiveness and feasibility of 3D-printed neurorehabilitation devices may differ in infrastructure-constrained settings. Printer type, printing materials, and fabrication processes were inconsistently reported across the included studies. We reported available details descriptively (Table 1) but were unable to synthesize fabrication methods across studies or provide implementation guidance.	10
Conclusions	21	This scoping review highlights the growing interest in 3D-printed rehabilitation technologies within neurorehabilitation, particularly in the development of orthoses for motor recovery [12,14]. While preliminary findings suggest that 3D printing may offer a flexible and cost-effective means of delivering personalized interventions, the current evidence base remains	10

SECTION	ITEM	PRISMA-ScR CHECKLIST ITEM	REPORTED ON PAGE #
		limited by small sample sizes and heterogeneous study designs [14]. To strengthen the field, future research should prioritize larger and more diverse populations; standardized protocols; and consistent use of validated, clinically meaningful outcome measures [54]. These efforts are essential to better assess the functional impact and real-world applicability of 3D-printed technologies in neurorehabilitation [54].	
**FUNDING**			
Funding	22	This research received no external funding. The authors did not receive any financial support for the conduct of this study.	10

JBI = Joanna Briggs Institute; PRISMA-ScR = Preferred Reporting Items for Systematic reviews and Meta-Analyses extension for Scoping Reviews.

* Where *sources of evidence* (see second footnote) are compiled from, such as bibliographic databases, social media platforms, and Web sites.

† A more inclusive/heterogeneous term used to account for the different types of evidence or data sources (e.g., quantitative and/or qualitative research, expert opinion, and policy documents) that may be eligible in a scoping review as opposed to only studies. This is not to be confused with *information sources* (see first footnote).

‡ The frameworks by Arksey and O'Malley (6) and Levac and colleagues (7) and the JBI guidance (4,5) refer to the process of data extraction in a scoping review as data charting.

§ The process of systematically examining research evidence to assess its validity, results, and relevance before using it to inform a decision. This term is used for items 12 and 19 instead of "risk of bias" (which is more applicable to systematic reviews of interventions) to include and acknowledge the various sources of evidence that may be used in a scoping review (e.g., quantitative and/or qualitative research, expert opinion, and policy document).

From: Tricco AC, Lillie E, Zarin W, O'Brien KK, Colquhoun H, Levac D, et al. PRISMA Extension for Scoping Reviews (PRISMAScR): Checklist and Explanation. Ann Intern Med. 2018;169:467–473. doi: 10.7326/M18-0850.
